# Pediatric Metastatic Pheochromocytoma and Paraganglioma: Clinical Presentation and Diagnosis, Genetics, and Therapeutic Approaches

**DOI:** 10.3389/fendo.2022.936178

**Published:** 2022-07-12

**Authors:** Mickey J. M. Kuo, Matthew A. Nazari, Abhishek Jha, Karel Pacak

**Affiliations:** ^1^ Medical Genetics Branch, National Human Genome Research Institute, National Institutes of Health, Bethesda, MD, United States; ^2^ Section on Medical Neuroendocrinology, Eunice Kennedy Shriver National Institute of Child Health and Human Development, National Institutes of Health, Bethesda, MD, United States

**Keywords:** pediatric, metastatic, pheochromocytoma, paraganglioma, clinical presentation, diagnosis, genetics, therapeutic approach

## Abstract

Although pediatric pheochromocytomas and paragangliomas (PPGLs) are rare, they have important differences compared to those in adults. Unfortunately, without timely diagnosis and management, these tumors have a potentially devastating impact on pediatric patients. Pediatric PPGLs are more often extra-adrenal, multifocal/metastatic, and recurrent, likely due to these tumors being more commonly due to a genetic predisposition than in adults. This genetic risk results in disease manifestations at an earlier age giving these tumors time to advance before detection. In spite of these problematic features, advances in the molecular and biochemical characterization of PPGLs have heralded an age of increasingly personalized medicine. An understanding of the genetic basis for an individual patient’s tumor provides insight into its natural history and can guide clinicians in management of this challenging disease. In pediatric PPGLs, mutations in genes related to pseudohypoxia are most commonly seen, including the von Hippel-Lindau gene (*VHL*) and succinate dehydrogenase subunit (*SDHx*) genes, with the highest risk for metastatic disease associated with variants in *SDHB* and *SDHA*. Such pathogenic variants are associated with a noradrenergic biochemical phenotype with resultant sustained catecholamine release and therefore persistent symptoms. This is in contrast to paroxysmal symptoms (e.g., episodic hypertension, palpitations, and diaphoresis/flushing) as seen in the adrenergic, or epinephrine-predominant, biochemical phenotype (due to episodic catecholamine release) that is commonly observed in adults. Additionally, PPGLs in children more often present with signs and symptoms of catecholamine excess. Therefore, children, adolescents, and young adults present differently from older adults (e.g., the prototypical presentation of palpitations, perspiration, and pounding headaches in the setting of an isolated adrenal mass). These presentations are a direct result of genetic determinants and highlight the need for pediatricians to recognize these differences in order to expedite appropriate evaluations, including genetic testing. Identification and familiarity with causative genes inform surveillance and treatment strategies to improve outcomes in pediatric patients with PPGL.

## Introduction

Pheochromocytomas and paragangliomas (PPGLs) are catecholamine-producing tumors that arise from chromaffin cells of the adrenal gland and extra-adrenal tissue, pheochromocytomas (PCC) and paragangliomas (PGL), respectively. Pediatric PPGLs have important differences compared to those in adults, a consequence of the more frequent genetic predisposition seen in the younger population (80% in pediatric patients compared to 40% in adults, see **Table 1**) (2, 4). In children with PPGL, disease is more likely to be extra-adrenal (30-60%), bilateral adrenal (12-48% of pediatric vs. 7-14% of adult patients), recurrent (13-38%), and multifocal (17-62%) (2, 4–8). The most common underlying genetic causes of pediatric PPGL are von Hippel-Lindau syndrome (VHL, 27-51%), mutations in succinate dehydrogenase subunit genes (collectively referred to as *SDHx*, 13-39% in *SDHB* and 8-10% in *SDHD*), multiple endocrine neoplasia type 2 (MEN-2, 0.6-10%), and neurofibromatosis type 1 (NF-1, 1-3%) (2, 4, 5, 7–11).

**Table 1:** Data on pediatric and adult PPGLs from studies directly comparing the two populations.

	Children	Adults
**Clinical Presentation** (1)		
Hypertension (sustained)	93%	68%
Hypertension (paroxysmal)	7%	26%
Normotension	0%	5%
Headache	95%	90%
Sweating	90%	92%
Tachycardia/dysrhythmias	35%	72%
Weight loss	15%	72%
**Location and Behavior** (2, 3)		
Unilateral PCC	12-22%	23-56%
Bilateral PCC	12-20%	9-26%
Solitary PGL	34%	22%
Multifocal	24-33%	5-14%
Metastatic	50%	29%
Synchronous metastases	26%	43%
Metachronous metastases	75%	57%
Recurrent primary tumors	30%	14%
**Biochemistry** (2)		
Adrenergic	7%	43%
Non-adrenergic*	93%	57%
**Genetics** (2, 3)		
Causal Gene** (*Cluster)*	70-84%	36-44%
*VHL (1B)*	27-32%	10-13%
*SDHB (1A)*	39-44%	17-26%
*SDHD (1A)*	10-16%	11-21%
*RET (2)*	3-4%	9-47%
*NF1 (2)*	1%	4%

*Non-adrenergic includes noradrenergic, dopaminergic, and non-secreting biochemical phenotypes. **Includes germline and somatic etiologies.

The underlying pathologic mechanisms that give rise to PPGL are heterogeneous and have been organized into clusters of genes by their effect on different intracellular pathways and gene expression profiling, including pseudohypoxia (cluster 1), kinase signaling (cluster 2), and Wnt signaling (cluster 3) (10, 12–15). The significance of this molecular taxonomy is not only descriptive but also helps to define different aspects of chromaffin cell metabolism that have direct clinical applications, such as the prototypical examples of VHL (cluster 1) and MEN-2 (cluster 2).

PCCs in patients with MEN-2 produce either only epinephrine or both norepinephrine and epinephrine (and their metabolites normetanephrine and metanephrine, respectively; collectively termed metanephrines) and have higher catecholamine levels due to increased expression of tyrosine hydroxylase, the rate-limiting enzyme in catecholamine synthesis (12). PCCs in VHL patients produce norepinephrine almost exclusively, related to decreased expression of the enzyme phenylethanolamine *N*-methyltransferase (PNMT) which converts norepinephrine to epinephrine (12). Furthermore, it was shown that expression of proteins involved in catecholamine secretion are differentially regulated between MEN-2 and VHL, as VHL-related PCCs show decreased expression of some secretory components, resulting in a less coordinated secretion system and therefore, continuous secretion of catecholamines in sharp contrast to paroxysmal release of catecholamines by MEN-2 PCCs (13). One study reported that, on average, norepinephrine-secreting PPGLs stored 1,760,000 picograms (pg) of norepinephrine/gram (g) tissue with 53% released each day, in contrast to epinephrine-secreting PPGLs that contained 3,801,000 pg of epinephrine/g tissue with only 5% released daily (16). Norepinephrine has a higher affinity for α_1_-adrenergic receptors, and epinephrine has a higher affinity for β_1_-adrenergic receptors, which broadly translates into more of an increased risk for hypertension in those with tumors that produce predominantly norepinephrine and more risk for tachycardia and arrhythmias in those tumors that produce predominantly epinephrine (17).

Taken together, these molecular and biochemical features are foundational for understanding the different clinical features in these patients, with paroxysmal hypertension occurring in the context of the adrenergic biochemical phenotype (increased epinephrine and metanephrine) of MEN-2 as compared to sustained hypertension with the noradrenergic biochemical phenotype (increased norepinephrine and normetanephrine) of VHL. Additionally, decreased expression of dopamine β-hydroxylase or tyrosine hydroxylase can result in a dopaminergic or biochemically silent (non-secreting) phenotype, respectively, which may both be seen from mutations in cluster 1 genes (18). Pediatric PPGL is more often due to pathogenic variants in cluster 1 genes (particularly *VHL*, *SDHx*, and *EPAS1*; at least 57-80% of patients with pediatric PPGL when somatic variants are included) and is thus more likely to have a non-adrenergic (noradrenergic, dopaminergic, or non-secreting) biochemical phenotype (93% of pediatric patients without increased plasma metanephrine vs. 57% in adults, see **Table 1**) (2, 4, 5, 7).

About 10-20% of PPGLs are diagnosed in pediatric patients (19), therefore, it is important for clinicians who care for pediatric patients to be aware of this treatable cause of hypertension, not only to alleviate the symptomatic burden of catecholamine excess, but also to make a timely diagnosis due to the risk of metastatic disease and consequent morbidity and mortality. Among adults under the age of 35 years with PPGL, many similarities to pediatric PPGL are seen (including hereditary, noradrenergic, and multifocal disease), and bilateral tumors were seen even more often than in children or in adults over the age of 35 years, suggesting that these tumors may have evaded clinical detection and were in fact present earlier in childhood (2), thus highlighting the need for clinicians to be aware of the possibility of PPGL in children and adolescents. Retrospective analysis from the Department of Defense Serum Repository found that in adults, biochemical evidence of elevated plasma metanephrines was seen at a median of 6.6 years (elevation above the upper reference limit [URL]) and 4.1 years (3 times above the URL) prior to diagnosis of PCC (20). Therefore, it is reasonable to suspect that pediatric PPGLs may go undetected for many years before diagnosis, putting these patients at risk not only for continued tumor growth and metastatic disease but also catastrophic complications if stored catecholamines in a clinically occult PPGL are suddenly released when provoked by surgery, induction of anesthesia, or as an adverse effect of medication.

While isolated PPGLs are often definitively treated by surgical removal, metastatic disease requires additional treatment modalities, such as chemotherapy, radiotherapy, targeted molecular therapies, or ablative therapies (21). Metastatic disease, defined by the presence of chromaffin tumor cells in tissues without chromaffin cells, is a major aspect of PPGL care and is itself largely genetically determined (1). Metastatic disease can occur in 12% of pediatric patients, in general, but may be up to 70% among *SDHB* mutation carriers, the second most commonly mutated gene in pediatric PPGL after *VHL* (19, 22, 23). The sites of involvement are most often bone, lymph nodes, liver, and lungs (though primary PGLs have been described in liver and lungs), which can result in significant impairment in quality of life and prognosis (24, 25). Metastases may be present at the time of initial diagnosis (synchronous) or later (metachronous or non-synchronous); in pediatric PPGL, metastases are less often synchronous as compared to adults, underscoring the importance of long-term surveillance for any child diagnosed with PPGL (2). In addition to those patients who harbor a mutation in *SDHB*, other established risk factors for metastatic disease include tumor size ≥ 5 cm, extra-adrenal PGL, dopaminergic phenotype (plasma 3-methoxytyramine higher than three times the URL), and a Ki-67 index > 3% (3, 26).

Finally, metastatic disease has direct implications in the approach to and management of pediatric PPGL, including imaging and treatment. Advances in functional imaging modalities with positron emission tomography/computed tomography (PET/CT) scans using different radionuclides provide insight into how patients from particular genetic backgrounds can receive an increasingly personalized approach to management of these challenging tumors. And while metastatic PPGL is incurable, knowledge of the molecular pathogenesis can direct treatments in some cases, informed by the cellular pathways disrupted by the genetic predisposition. For these reasons, the genetic background may guide management of the pediatric PPGL patient, and genetic testing is essential to providing optimal care for these patients.

## Clinical Features

PPGLs are estimated to occur at an incidence of 0.57 per 100,000 person-years, of which, up to 20% of PPGLs are diagnosed in pediatric patients, at an average age of 11 years (19, 22, 23, 27). Most pediatric patients are symptomatic (around 90%), and hypertension is the most common presentation of pediatric PPGL (64-93%), which causes pediatric hypertension in up to 1% of cases (7, 8, 22). Therefore, one should suspect PPGL in pediatric patients with sustained hypertension, with headache (39-95%), diaphoresis (90%), palpitations (53%), and signs/symptoms of mass effect or as an incidental mass (30%) (**Table 1**) (8, 11, 22).

Pediatric hypertension is defined as an auscultatory blood pressure > 130/80 mmHg for children ages 13 years and above or ≥ 95^th^ percentile for age, sex, and height for children ages 1 to 12 years, on more than 3 occasions, according to the most recent Clinical Practice Guideline from the American Academy of Pediatrics (28). Pediatric hypertension can be further complicated by hypertensive emergency in patients with catecholamine excess, such as retinopathy, resulting in visual disturbances, and hypertensive encephalopathy, resulting in seizures and disorientation (22, 29). Hypertrophic and dilated cardiomyopathy can arise as sequelae of hypertension in pediatric patients with PPGL; among pediatric patients with dilated cardiomyopathy, surgical excision of their tumors led to resolution of hypertension and improved cardiac function (8, 30).

Tachycardia and dysrhythmias, by contrast, are seen more often in adults, which may be related to the relatively higher proportion of cluster 2 mutations in adults resulting in stimulation of cardiac β_1_-adrenergic receptors by epinephrine (22). Stimulation of β-adrenergic receptors by epinephrine in cluster 2 mutations results in hepatic glycogenolysis and gluconeogenesis, which may account for the higher proportion of adults as opposed to children who present with elevated fasting glucose levels (22, 31).

In addition to the signs and symptoms noted above, other findings related to catecholamine excess may be non-specific and include pallor, orthostatic hypotension and syncope, shortness of breath, abdominal pain, nausea, vomiting, constipation, diarrhea, hyperglycemia, polyuria and polydipsia, anxiety, behavioral symptoms, worsening performance in school, and ADHD (9, 30, 32). Interestingly, among pediatric patients harboring an *SDHB* mutation, sweating was significantly associated with earlier development of metastatic disease (*p* = 0.0073), and for those without metastases at initial diagnosis, patients with tumor pain developed metastases at an earlier interval than those without tumor pain (*p* = 0.0088) (23).

Presenting signs and symptoms may also be related to mass effect of the growing tumor, and patients may present with a palpable abdominal mass on physical examination (30). Abdominal masses may present with abdominal pain and distension or back pain, and bladder masses may result in hematuria or symptoms with voiding (11, 32). Parasympathetic head and neck PGLs (HNPGLs) would not be expected to produce catecholamines, and the clinical presentation may be related to mass effect on cranial nerves and other local structures, resulting in hearing loss, tinnitus, hoarseness, dysphagia, cough, pain, or feeling of fullness in the neck (32). As in adults, PPGLs may also be found as an incidental mass on imaging studies performed for other indications (8, 11). For patients with clinical findings of or genetic predisposition to PPGL, biochemical evaluation is indicated.

## Biochemical Foundations

**Figure 1:**
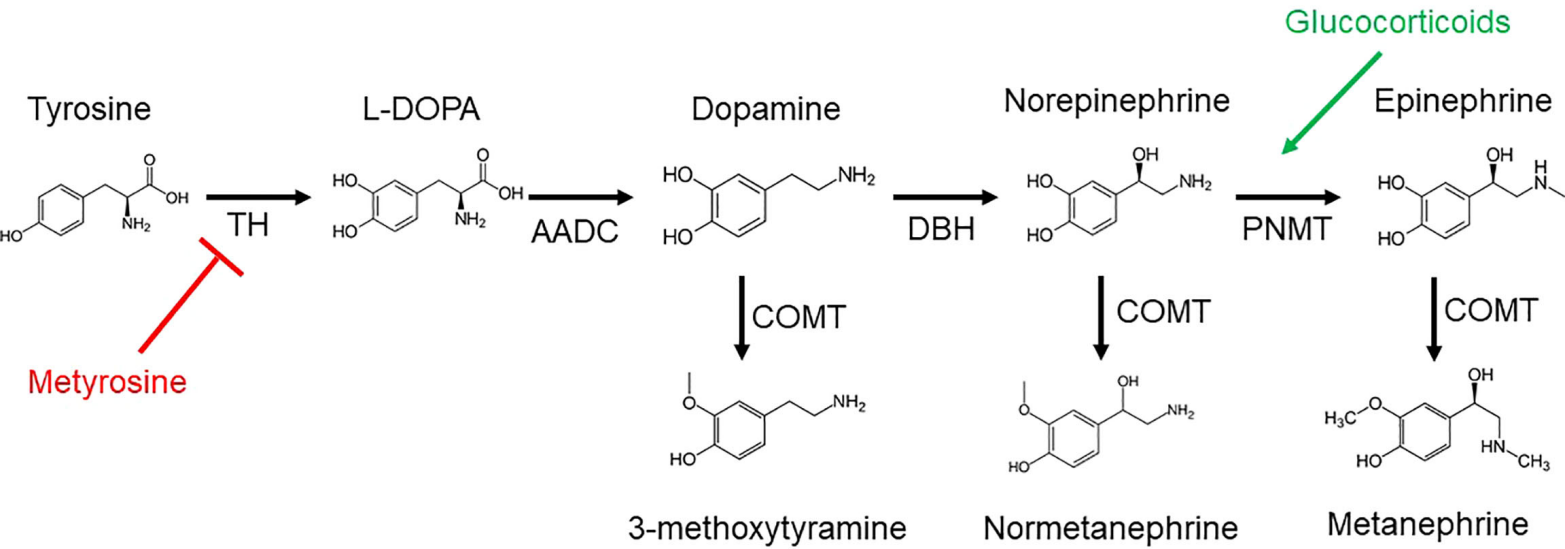
Catecholamines and metanephrines are derived from tyrosine by the enzyme tyrosine hydroxylase (TH), resulting in L-3,4-dihydroxyphenylalanine (L-DOPA). Aromatic L-amino acid decarboxylase (AACD) generates dopamine from L-DOPA. Dopamine β-hydroxylase (DBH) acts on dopamine to produce norepinephrine, followed by the enzyme phenylethanolamine *N*-methyltransferase (PNMT) to yield epinephrine. Norepinephrine and epinephrine are *O*-methylated by catechol-*O*-methyltransferase (COMT) to produce normetanephrine and metanephrine, respectively. The corresponding *O*-methylated metabolite of dopamine is 3-methyoxytyramine (3-MT). Metyrosine inhibits (red) TH. Glucocorticoids stimulate (green) PNMT.

The biosynthesis of catecholamines, metanephrines, and 3-methoxytyramine (3-MT, the *O*-methylated metabolite of dopamine), as derivatives of tyrosine, is shown in **Figure 1**. Plasma free metanephrines should be measured, as this highly sensitive test can help to rule out PPGL in children, as in adults (33). Eisenhofer et al. found that the optimal combination of diagnostic sensitivity (97.9%) and specificity (94.2%) was achieved by using age-nonspecific URLs for metanephrine (446 picomoles/liter [pmol/L]) and 3-methoxytyramine (107 pmol/L) with age-specific URLs for normetanephrine, increasing from age 5 years (542 pmol/L) to age 65 years (1092 pmol/L) as modeled by the equation *URL_NMN_* = (2.07 × 10^-3^ × *age* ^3^) + 545 (pmol/L) (**Table 2**) (34). Although plasma fractionated metanephrines are the test of choice in children as in adults, urinary free metanephrines may be considered without much loss of sensitivity (97.9% for plasma free metanephrines and 93.4% for urinary free metanephrines), which may be preferable to avoid venipuncture in some children (35).

**Table 2:** Age-specific upper reference limits for normetanephrine.

	Pediatric			Adult		
Age (years)	5	12	19	35	50	65
Normetanephrine	542	549	559	634	804	1092

Units expressed as pmol/L. Values between 5 and 65 years of age were interpolated based on the equation in reference (31).

Dopamine-producing PPGLs show elevations of 3-MT; thus, plasma 3-MT should be used to increase the diagnostic sensitivity in PGLs, especially for HNPGLs (22.1% to 50%) using a cut-off of 0.1 nanomoles/liter (nmol/L) (36). Urinary dopamine reflects renal metabolism and is not helpful to identify dopaminergic PPGL (36). Although testing for 3-MT is of more limited clinical availability than that of metanephrines, it is helpful to assess risk of metastatic disease, as plasma elevations above 0.2 nmol/L had a 57% sensitivity and 85% specificity for metastatic disease (37).

Chromogranin A is a biomarker of neuroendocrine tumors, including PPGL, and has been shown to increase the sensitivity of diagnosis when used in conjunction with metanephrines, especially in patients with *SDHB*-related PCC or sympathetic PGL, though its performance was not as good in patients with HNPGL (38). As its production is independent of the catecholamine metabolism pathway, it is a helpful adjunct to detect non-secreting tumors.

Care must be taken to reduce false-positive biochemical laboratory results. This can be achieved by avoiding circumstances that provoke an adrenergic response at the time of collection. For this reason, the patient should be placed in a supine position with venipuncture performed 20-30 minutes before biochemical labs are obtained – as both non-supine position and venipuncture may lead to catecholamine release (32). Medications that can artifactually increase plasma or urinary fractionated metanephrines include acetaminophen, α-methyldopa, tricyclic antidepressants, monoamine oxidase inhibitors, sympathomimetics, catecholamine reuptake inhibitors, mesalamine/sulfasalazine, phenoxybenzamine, levodopa, anesthetics, neuromuscular blockers, antiemetics, linezolid, peptide hormones, steroids, cocaine, and opioids (17, 39). Glucocorticoids potentiate catecholamine biosynthetic enzymes and should be avoided in patients with PPGL due to risk of causing PCC crisis (40). Therefore, when able, these medications should be held before biochemical labs are obtained.

Cellular consequences of cluster 1 mutations converge on hypoxia-inducible factor 2α (HIF-2α), a transcription factor that dimerizes with HIF-1β and mediates downstream effects such as blocking the glucocorticoid-induced expression of PNMT (41). Gain-of-function mutations in *EPAS1* (encoding for HIF-2α) contribute to aberrant neuroendocrine-to-mesenchymal transition which gives rise to PPGLs and contributes to their metastatic behavior (41). Biochemically, this translates to the observation that PNMT and other biosynthetic enzymes are down-regulated in patients with cluster 1 mutations, such that the biochemical phenotype is either noradrenergic, dopaminergic, or non-secreting, but not expected to be adrenergic.

Finally, the metabolic role of succinate dehydrogenase in the tricarboxylic acid (TCA) cycle and electron transport chain is related to diagnostic imaging and may impact treatment. Mutations in TCA cycle genes (cluster 1A) result in decreased function of these metabolic enzymes and increase dependency on glycolysis for energy production (15). Radiologically, this correlates with higher uptake of ^18^F-fluorodeoxyglucose (^18^F-FDG) on PET imaging for tumors of a cluster 1 genetic background when compared with cluster 2 (42). In addition to its role in the conversion of succinate to fumarate in the TCA cycle, succinate dehydrogenase also plays a crucial role as complex II in the mitochondrial electron transport chain, using reducing equivalents of FADH_2_ to ultimately generate ATP (43, 44). Defects in SDH subunit genes (i.e., *SDHx*) may increase the cell’s dependence on NADH oxidation through complex I, resulting in increased availability of NAD^+^ to be used by poly (ADP-ribose) polymerase (PARP) in base excision repair of DNA (43). Therefore, *SDHx* mutations increase chemoresistance to genotoxic agents by stimulating DNA repair mechanisms via PARP. For these reasons, PARP inhibition may deprive these tumors of this protective mechanism and play a role in personalized treatment tailored by genetics (43).

## Genetics

### Overview

Pediatric PPGLs are often found to have a genetic cause, which is most commonly due to a germline mutation in one of the following susceptibility genes: *VHL*, *SDHx*, *RET*, and *NF1*. Genes related to pseudohypoxia in cluster 1 are subdivided into cluster 1A with TCA-cycle genes, including the *SDHx* genes, and cluster 1B related to hypoxia signaling, most significantly *VHL* and *EPAS1*. The other two important genetic causes of pediatric PPGL, *RET* and *NF1*, are in cluster 2, related to kinase signaling (15, 26).

Genetic syndromes predisposing to PPGL are all inherited in an autosomal dominant manner, but with the caveat that pathogenic variants in *SDHD* (as well as *SDHAF2* and *MAX*) increase PPGL susceptibility when paternally inherited (18). However, identifying a maternally inherited *SDHD* variant has implications for screening extended family members and subsequent generations. While a family history of PPGL is often seen in pediatric patients with PPGL, the absence of a family history should not disincline clinicians from referring for genetic counseling and testing, as one must consider the possibility of *de novo* mutations. All patients with PPGL should be referred for genetic counseling and evaluation (1).

### Succinate Dehydrogenase Subunit Defects

Mutations in all four subunits of succinate dehydrogenase (subunits A, B, C, and D) and assembly factor gene *SDHAF2* have been described in PPGL. In addition to sequence variants and copy number changes contributing to the genetic pathogenesis of *SDHx* variants, disease resulting from epigenetic alteration of *SDHC* by increased promoter methylation (i.e., epimutation) resulting in decreased expression have also been described, especially in PGLs and gastrointestinal stromal tumors (GISTs) (45, 46). Accumulation of succinate results in impaired prolyl hydroxylation of HIF-2α, resulting in the pseudohypoxic expression pattern seen for these cluster 1A genes, as well as other epigenetic effects on gene expression (47). In addition to PPGLs, other tumors, including renal cell carcinoma (RCC), GIST, pituitary adenoma, pulmonary chondroma (as part of Carney triad, which also includes PGL and GIST), and papillary thyroid cancer, as well as thyroid nodules have been reported in patients with *SDHx* mutations or *SDHC* epimutation; estimated penetrance of RCC in *SDHx* carriers is 2-3% (mostly *SDHB*), but 85% of GISTs diagnosed in childhood are related to *SDHx* mutations (4, 7, 48). No additional imaging is recommended to screen for these tumors in asymptomatic *SDHx* carriers (48). While genotype-phenotype correlations have been explored in *SDHx* genes, particularly for *SDHD* and *SDHB*, variable expressivity is seen, such that even among family members with the same variant, the sites and extent of disease and presence of metastases may vary (48).

Mutations in *SDHB* (on chromosome 1p36.13) are the most well-established genetic risk factor for metastatic PPGL. In a study from the National Institutes of Health that assessed 125 patients with metastatic PPGL, 32 presented before 20 years old, of which 23 (71.9%) were found to have a germline mutation in *SDHB* (49). Although the penetrance of PPGL in *SDHB* was initially thought to be higher, a Dutch study using data ascertained from clinical genetics centers (as opposed to PPGL tertiary care centers) found that the penetrance was closer to 21% by age 50 and 42% by age 70 (50). The risk of metastatic disease for *SDHB* carriers with PPGL is reported to be about 34-71%; in a study of 64 pediatric PPGL patients with *SDHB* mutations reported by Jochmanova et al., 70% developed metastases at a median age of 16 years (those patients being diagnosed at a median age of 12 years). Among those with metastases, only 19% had synchronous metastases, with the highest risk of metastatic disease within the first 2 years after diagnosis and again between 12-18 years after diagnosis (23), highlighting the critical role of lifelong surveillance and monitoring in these patients. Recurrent disease was seen in 20% of these patients, at a median age of 16 years (median time from diagnosis to recurrence was 2 years); recurrence was more often as sympathetic PGL (sPGL) than PCC (85% compared to 38%, respectively). Tumor size also played an important role in the timing of metastases as patients with tumors ≤ 5 cm developed metastatic disease at a median interval of 7 years compared to 2 years in those with tumors > 5 cm (23). All patients who died (12.5%) had metastatic disease, surviving for a median duration of 7.5 years after diagnosis of metastatic disease; no differences in the time to death were seen by comparison among the 5 most common mutations (one missense, two premature nonsense mutations, and two exonic deletions) (23). A genetic analysis of two European datasets (Germany and Great Britain) comparing different types of mutations in *SDHB* carriers with PPGL found that truncating mutations in *SDHB* seemed to confer a higher risk of PPGL than missense variants (62% vs. 38%), which remained statistically significant after removing prominent founder variants from one of the datasets; malignancy was also more common in those with truncating variants (62% vs. 38%), but no significant difference was seen for HNPGL between these different *SDHB* variants (47% for truncating vs. 53% for missense variants). No difference in age of diagnosis was seen between truncating and missense *SDHB* variants (46).

Mutations in *SDHD* (on chromosome 11q23.1) have a higher penetrance than those in *SDHB* (> 80%), and the autosomal dominant inheritance pattern is modified by maternal imprinting such that the disease is usually inherited from the paternal allele (46). Cases involving confirmed maternal inheritance are rare and involve additional genetic events, such as post-zygotic loss of chromosome 11 material as demonstrated in one study of 20 such patients with only one who was confirmed to have PPGL (35 years woman with PCC) (51). *SDHD* mutations are commonly seen in HNPGLs, but thoracic PGLs and PCCs are also seen (4, 18). Metastatic risk in *SDHD* is reported to be around 15-29% (26). In a study of 32 pediatric PPGL patients who developed metastatic disease, the three patients with a primary HNPGL were all identified to have a *SDHD* mutation (49), however in three other studies of pediatric PPGL patients, 17 of 177 patients, 5 of 88 patients, and 2 of 25 patients were found to have *SDHD* mutations, and only 2 of 17 later developed metastatic disease (4, 5, 7).

Pathogenic variants in *SDHC* (on chromosome 1q23.3) and *SDHA* (on chromosome 5p15.33) contributing to PPGL are much less common, with estimates of lifetime penetrance for PPGL at 8.3% and 1.7%, respectively (52). While metastatic disease risk is estimated to be low for *SDHC* mutations, metastatic risk in *SDHA* is 30-66% (26). Bausch et al. reported that among 177 pediatric PPGL patients, there was one patient who carried pathogenic variants in each of these genes: a 12 years male with head and neck PGL (*SDHC*) who did not develop metastases and a 15 years male with PCC (*SDHA*) who died within 13 years of follow-up (4). Interestingly, SDHC and SDHD share structural (anchoring the SDH complex to the inner mitochondrial membrane) and bioenergetic roles (the SDHC-SDHD dimer transfers electrons from iron-sulfur clusters of SDHB to ubiquinone in the electron transport chain) as well as both commonly presenting as head and neck PGLs (18, 44).

International consensus guidelines were published for PPGL screening in asymptomatic *SDHx* carriers (*SDHA*, *SDHB*, *SDHC*, and paternally-inherited *SDHD* variants), which should start in childhood at age 6-10 years for *SDHB* carriers and otherwise at 10-15 years and include clinical evaluation with symptom assessment, physical examination, and measurement of blood pressure; biochemical testing with plasma or urine metanephrines; and magnetic resonance imaging (MRI) studies of the head and neck as well as of the thorax, abdomen, and pelvis (48). If the initial evaluation is negative, follow-up should include annual clinical evaluation, biochemical testing every 2 years, and MRI every 2-3 years. Functional imaging by PET/CT was included for initial screening in adults, but no recommendation was made for screening by functional imaging in children (48).

### Von Hippel-Lindau Syndrome

Due to pathogenic variants in *VHL* (on chromosome 3p25.3), VHL syndrome is due to loss of function of this tumor suppressor gene that encodes for an E3 ubiquitin ligase that targets HIF-2α for degradation (53). *VHL* is a cluster 1B gene and is associated with a noradrenergic biochemical phenotype. PPGL develops in about 10-25% of VHL patients, typically presenting as PCC, though sympathetic and parasympathetic PGLs can also be seen, and the risk of metastatic disease is 5-8% (26, 54). Other tumors seen include angiomas and hemangioblastomas of the retina and central nervous system, renal and pancreatic cysts, RCC, endolymphatic sac tumor, pancreatic neuroendocrine tumor, and papillary cystadenoma of the epididymis or broad ligament (54). Genotype-phenotype correlations have been reported in VHL: type 2 VHL, associated with higher risk of PCC, is usually due to missense variants, whereas type 1 VHL, with a lower risk for PCC, includes truncating variants and exon deletions as well as missense mutations (53, 54). In about 20% of cases, patients will have a *de novo* mutation; interestingly, a study of Spanish PPGL patients found a much higher *de novo* rate of 60% overall and 50% for the pediatric group, though the authors attributed this to sample size (29, 54). VHL is the most common genetic cause of pediatric PPGL and tends to present the earliest at an average age of 11-12 years (4, 5, 7, 8, 22), in contrast to those syndromes arising from pathogenic variants in cluster 2 – specifically, MEN-2 (14-20 years) and NF1 (16-17 years); PCC may be the first manifestation of VHL disease (4, 5, 7, 8, 22). While screening recommendations for VHL may vary, PCC has been identified in a girl as young as 2 years, so measurement of blood pressure at each medical appointment and annual plasma free metanephrines or 24-hour urine fractionated metanephrines starting as early as 2 years but no later than 5 years has been proposed (54). Other aspects of screening for VHL-associated tumors, including retinal examination, audiology evaluation, and MRI studies are not discussed here but should be included as part of the comprehensive care for these patients.

### *EPAS1* Gain-of-Function Syndrome

One notable exception to the general rule of germline susceptibility is Pacak-Zhuang syndrome (PZS), characterized by polycythemia, PPGL, and duodenal somatostatinoma (55, 56). PZS is usually due to post-zygotic somatic mutations (rarely associated with germline inheritance) in the *EPAS1* gene (on chromosome 2p21), encoding for the transcription factor HIF-2α (55, 56). This syndrome was first described in two female patients with congenital polycythemia and onset of PGL during adolescence (55, 56). In PZS, gain-of-function mutation results in impaired prolyl hydroxylation of HIF-2α, increasing its stability by interfering with ubiquitination by the VHL protein (pVHL). Transcriptional effects include expression of erythropoietin, resulting in polycythemia, and vascular endothelial growth factor (VEGF), contributing to a pro-angiogenic phenotype, and resulting in decreased expression of PNMT, characteristic of cluster 1 mutations (41, 55). This increased stability also affects differentiation of neural crest progenitor cells that may promote metastatic behavior such as facilitating the neuroendocrine-to-mesenchymal transition (41). Pamporaki et al. reported that 4 of 92 pediatric patients (4.3%) compared to 5 of 519 adult patients (1.0%) with PPGL were found to have a somatic mutation in *EPAS1* from tumor tissue, emphasizing how frequently cluster 1 mutations, even post-zygotic mutations, are seen in the pediatric population (2). In another cohort, Redlich et al. identified 1 of 88 (1.6%) pediatric PPGL patients with an *EPAS1* somatic mutation and clinical features of PZS (including abdominal PGL) but without evidence of metastatic disease (7). A study of 7 patients with PZS showed that PGLs were multiple and recurrent in all patients but only 2 of 7 (29%) had metastatic disease (57). These patients highlight the benefit of somatic – in addition to germline – genetic testing when patient tumor tissue is available, as it may provide additional clues to the underlying etiology and raise awareness of associated clinical features and risk of multiplicity, recurrence, and malignancy.

### Neurofibromatosis Type 1

Mutations in the *NF1* gene (encoding for neurofibromin), located on chromosome 17q11.2, are responsible for neurofibromatosis type 1. Though both are in cluster 2, in contrast to the *RET* gene, *NF1* is a tumor suppressor that negatively regulates the RAS-MAPK signaling pathway. Partly due to historical challenges in sequencing related to the size of this gene, clinical diagnostic criteria were developed at the National Institutes of Health and include the presence of café-au-lait macules, axillary or inguinal freckling, neurofibromas, optic glioma, Lisch nodules, distinctive bone lesions, and an affected first-degree relative (58). While an often-cited retrospective analysis showed that PCCs were seen in 0.1 to 5.7% of patients with NF-1, 3.3 to 13.0% of NF-1 patients were found to have PCC on autopsy (59). A prospective study of adults with NF-1 identified PCC in 7.7% of patients, suggesting that these tumors are often found incidentally in asymptomatic patients (60). Unilateral adrenal disease is most common (78-84%), with bilateral disease seen in only 9.6 to 16.6% of patients with PCC, and metastatic disease is seen in up to about 11.5% of patients, though these estimates are derived mostly from adult NF-1 patients. Extra-adrenal locations include the organ of Zuckerkandl, abdominal sympathetic chain, and bladder (59–61). Adrenal findings in NF-1 include benign adrenal nodules, adrenal hyperplasia, and PCCs, which may be found histologically as composite tumors (with ganglioneuroma, ganglioneuroblastoma, neuroblastoma, and mixed neuroendocrine-neural tumor); GIST, renal oncocytoma, and carcinoid tumors have also been reported in NF-1 (59, 60). By contrast, pediatric PPGL in NF-1 is far less commonly seen, with reports indicating that these patients comprise only 1-3% of pediatric PPGL cohorts and present as teenagers (15-17 years), though 3 of 8 patients developed metastatic disease (one of which had microscopic residual tumor on histopathological analysis) (2, 4, 7). An important consideration is that up to 50% of the cases of NF–1 arise *de novo*, another reason why the absence of a family history *per se* should not preclude NF–1 from the differential diagnosis in the presence of suggestive clinical findings (59).

### Multiple Endocrine Neoplasia Type 2

MEN-2 is due to pathogenic variants in the *RET* gene (on chromosome 10q11.21), a cluster 2 proto-oncogene encoding for a receptor tyrosine kinase that plays an important role in the development of neural crest cells (62). MEN-2A is associated with the combination of medullary thyroid cancer (MTC, > 90%), hyperparathyroidism (15-30%), and PCC (57%) and accounts for up to 95% of MEN-2 (including familial MTC [FMTC], a variant of MEN-2A), whereas MEN-2B constitutes about 5% of MEN-2 and is characterized by early and aggressive MTC, PCC (50%), mucosal neuromas, and a marfanoid habitus (63, 64). As a proto-oncogene, disease arises due to gain-of-function mutations in *RET*, often due to missense mutations at cysteine residues, such as at codons 609, 618, and 620 in exon 10 or 634 in exon 11, which result in MEN-2A (63, 64). In contrast, about 95% of individuals with MEN-2B have the p.M918T mutation in exon 16, and about 75% of patients have disease resulting from a *de novo* mutation (63, 64). Genotype-phenotype correlations are particularly important in MEN-2, and the 2015 Revision of the Guidelines for Management of Medullary Thyroid Cancer by the American Thyroid Association has classified *RET* variants into three risk categories on the basis of risk for MTC, which for the “high” and “highest” risk categories also correspond to those mutations for the greatest risk of PCC: "highest" (only the p.M918T mutation), "high" (p.A883F and p.C634 mutations), and "moderate" (other mutations) (63). With mutations of the cysteine residue at codon 634, the risk of PCC, hyperparathyroidism, and cutaneous lichen amyloidosis is higher, and specifically for the p.C634R mutation, there is a higher risk of metastatic MTC at diagnosis (64). For *RET* mutations at codons 918, 883, and 634, screening for PCC should begin at age 11 years, but for those moderate risk mutations, screening may begin at age 16 years (63). PCCs in MEN-2 are often multicentric and bilateral, developing in the setting of diffuse nodular adrenal medullary hyperplasia (63). Metastatic risk of PCC is < 5% (26). Among 6 studies of pediatric PPGL patients with MEN-2 due to a *RET* mutation, ten patients were identified (representing 0.6-3.3% of each cohort except for 13.0% in one study), all with PCC Age at PCC diagnosis ranged from 14-20 years; eight patients had bilateral or recurrent disease, and no patients were reported to have metastatic disease (2, 4, 5, 7, 8, 29). Though not discussed here, other aspects of MEN-2, including hyperparathyroidism and MTC, require clinical consideration and management (63).

## Imaging

Anatomic imaging is essential to the diagnosis for any patient being evaluated for PPGL and is typically performed by CT or MRI. In pediatric patients, high signal-intensity T2-weighted MRI is preferable over CT to reduce radiation exposure and may be better suited to identify extra-adrenal tumors, invasion into the spinal canal, and involvement of major vessels (2, 9, 11, 65). Children may not tolerate MRI as well as adults and may require sedation, so clinicians should be mindful regarding the choice of sedative so as not to cause precipitous catecholamine release; medications such as opioids (except for fentanyl) can cause histamine release, which may stimulate catecholamine secretion (66).

Anatomical imaging is used for tumor localization and detection of metastases after positive biochemical testing but also for screening, as non-functional tumors may be negative on biochemical evaluation, such as in patients with *SDHx* mutations or those with parasympathetic PGLs arising in the head and neck (48). For follow-up imaging in the setting of metastases, CT and MRI can be complementary, as CT is better for visualizing lung lesions, and MRI is better for liver lesions (26).

Functional imaging provides insight into the molecular features of PPGLs by use of different radiopharmaceuticals, which is especially informative in metastatic disease. *“Theranostics”* refers to this unification of *thera*peutic and diag*nostic* utility with the same radiopharmaceutical platform, for example using ^123^I-labeled metaiodobenzylguanidine (MIBG) scintigraphy to identify PPGLs which can then be treated by ^131^I-MIBG (67). MIBG is an analog of norepinephrine and enters tumor cells via the norepinephrine transporter (42, 68). Medications such as over-the-counter cough and cold treatments, calcium channel blockers, labetalol, and tricyclic antidepressants can interfere with MIBG uptake and should be avoided before treatment. Special preparation is also required to reduce accumulation of radioiodine in the thyroid (68). While highly specific, MIBG may not be as sensitive as other functional imaging modalities, particularly in hereditary PPGL, and especially in those with *SDHx* mutations. Additionally, sensitivity is higher for PCC than PGL (88% vs 67%, respectively), and in metastatic PPGL, per-lesion sensitivity is < 60% (42). Despite these limitations in the pediatric population with metastatic PPGL, MIBG may still have a useful theranostic role in patients where ^123^I-MIBG avidity is observed in all lesions.

**Figure 2:**
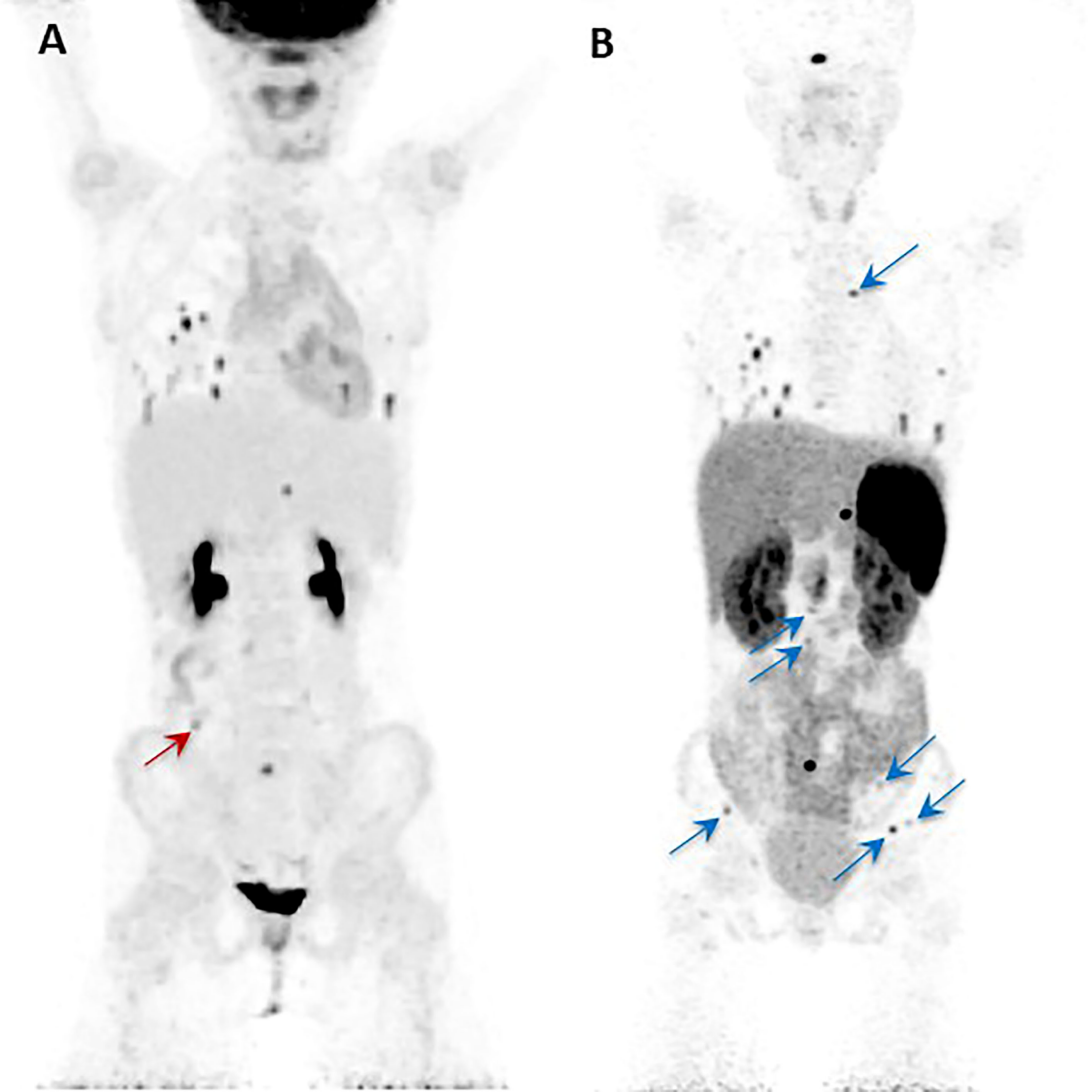
Anterior maximal intensity projection (MIP) images of the ^18^F-FDG PET/CT **(A)** and ^68^Ga-DOTATATE PET/CT **(B)** studies of a 10-year-old *SDHB* positive girl. She was diagnosed initially with metastatic disease at the age of 8 years. Her right paraaortic, retroperitoneal primary paraganglioma was surgically resected. On presentation to our institution, the progression of her disease was demonstrated by metastatic lesions in bone, lungs, and abdomen as shown in the images **(A, B)**. The single red arrow on image **(A)** indicates the one lesion (abutting bowel) localized by ^18^F-FDG PET/CT that is not visualized by the ^68^Ga-DOTATATE PET/CT. Similarly, all the additional lesions (transverse process of T4 spine, L2-L5 vertebral bodies, left ilium, and left and right iliac wings) localized by the ^68^Ga-DOTATATE PET/CT (blue arrows) are not visualized by ^18^F-FDG PET/CT **(B)**. This figure was adapted from the figure that was initially published as Figure 2 by Jha et al. (70).

The other relevant application of the theranostic paradigm is for tumors expressing somatostatin receptors – especially type 2 (SSTR2) – that bind to somatostatin analogs (SSAs) linked to a chelator (DOTA) for the radionuclide. Typically, ^68^Ga- or ^64^Cu-DOTA-SSA is used for imaging, and ^90^Y- and ^177^Lu-DOTA-SSAs deliver the radiation dose to the sites of tumors, with the latter referred to as peptide receptor radionuclide therapy (PRRT) (42, 69). ^68^Ga-DOTATATE is commonly used since DOTATATE binds preferentially to SSTR2 over other somatostatin receptors, as opposed to DOTATOC or DOTANOC (42). ^68^Ga-DOTATATE is particularly useful for cluster 1A mutations (especially *SDHx*), HNPGLs, metastatic PPGL, and pediatric PPGL (42). Among nine pediatric patients with *SDHx* mutations, both ^18^F-FDG and ^68^Ga-DOTATATE detected lesions in all of the patients but ^68^Ga-DOTATATE had superior sensitivity on a per-lesion basis (94% compared to 79% on ^18^F-FDG) and was also more sensitive than anatomic imaging by CT or MRI with contrast enhancement (74%). ^68^Ga-DOTATATE was especially superior to CT/MRI for mediastinal lesion detection and superior to ^18^F-FDG for detection of adrenal and liver lesions, though ^18^F-FDG and CT/MRI outperformed ^68^Ga-DOTATATE for detection of other abdominal lesions (**Figure 2**) (70). These data offer a compelling case for performing ^68^Ga-DOTATATE PET/CT or ^68^Ga-DOTATATE PET/MRI with contrast enhancement to detect such abdominal lesions. Currently both functional and anatomic imaging are required for staging and for assessing treatment response in pediatric metastatic PPGL patients and, therefore, simultaneous PET/MRI may be considered in this cohort due to decreased radiation exposure, fewer instances requiring sedation or general anesthesia, fewer appointments, and simultaneous imaging with two advanced diagnostic imaging techniques (whole-body PET and MRI) (71). Recommended imaging modalities for different types of PPGL based on genetic and clinical features are summarized in **Table 3**. A 2015 meta-analysis by Han et al. pooled patients of unknown genetic background, finding a significantly superior detection rate of ^68^Ga-DOTA-conjugated somatostatin receptor-targeting peptide (^68^Ga-DOTA-SST) PET (93%) as compared to other functional imaging modalities (72). Therefore, in cases where a genetic etiology is not known, ^68^Ga-DOTATATE PET may be performed.

**Table 3:** Functional imaging recommendations for pediatric PPGL.

PPGL Type	Recommended Imaging Modality	Alternative
Cluster 1A	^68^Ga-DOTATATE PET/CT	^18^F-FDG PET/CT
Metastatic		
Cluster 1B	^18^F-DOPA PET/CT	^123^I-MIBG scintigraphy
Cluster 2		
Sporadic PCC		
HNPGL	^68^Ga-DOTATATE PET/CT	^18^F-DOPA PET/CT

Recommended imaging modalities for different PPGL types. Note that ^123^I-MIBG scintigraphy should also be considered for metastatic disease if ^131^I-MIBG therapy would be performed.

^18^F-fluorodihydroxyphenylalanine (^18^F-FDOPA) enters cells via the large neutral amino acid transporter (LAT-1) and enters into the catecholamine synthesis pathway (42). Preparation for ^18^F-FDOPA PET/CT involves fasting and administration of carbidopa to block decarboxylation of DOPA to dopamine, improving uptake in target tissue. There are no known interfering medications (42). ^18^F-FDOPA PET/CT is particularly helpful for imaging PPGLs in clusters 1B (VHL and PZS) and 2 (MEN-2 and NF-1) and has an advantage over MIBG in that normal adrenal tissue has lower uptake, increasing the sensitivity to detect nonmetastatic PCC (94% in patients of known genetic background and up to 100% in patients with apparently sporadic nonmetastatic PCC); indeed, the detection of metastatic PPGL may also vary on the basis of the genetic background with greater sensitivity in non-*SDHx* PPGL (42, 73).

^18^F-FDG is a radiolabeled form of glucose and is taken up by glucose transporters (particularly GLUT-1) and enters into the glycolytic pathway (42). Preparation for ^18^F-FDG involves fasting, controlling hyperglycemia, and avoidance of ambient cold temperature to prevent artifacts from glucose metabolism in thermogenic brown fat (42, 74). The sensitivity of ^18^F-FDG in detection of metastatic PPGL is superior to MIBG (usually > 80%), particularly in *SDHx* patients, with sensitivity in metastatic disease for *SDHx* patients ranging from 83-92% compared to 62% for non-*SDHx* (42). Furthermore, while there are no studies of direct comparisons, GIST appears to be better appreciated on ^18^F-FDG compared to ^68^Ga-DOTATATE, and therefore, some *SDHx* patients would benefit from ^18^F-FDG in addition to ^68^Ga-DOTATATE PET/CT scan when GIST is suspected (75, 76).

## Management

After biochemical studies and tumor localization by imaging, definitive treatment of PPGL should be addressed. In addition to surgery of the primary tumor, when appropriate, patients with metastatic disease require other treatment modalities to address disease at sites of metastasis, such as chemotherapy, radiotherapy, ablative therapy, or other targeted therapies, such as somatostatin analogs or small molecule inhibitors (21). Clinical trials for pediatric patients with advanced/metastatic PPGL that are recruiting, active, or approved are listed in **Table 4**.

**Table 4:** Clinical trials for metastatic PPGL therapies in pediatric patients that are recruiting, active, or approved.

Intervention	Class	NCT Number	Title
Belzutifan	HIF-2α inhibitor	NCT04924075	Belzutifan/MK-6482 for the Treatment of Advanced Pheochromocytoma/Paraganglioma (PPGL) or Pancreatic Neuroendocrine Tumor (pNET) (MK-6482-015)
DFF332	HIF-2α inhibitor	NCT04895748	DFF332 as a Single Agent and in Combination with Everolimus & Immuno-Oncology Agents in Advanced/Relapsed Renal Cancer & Other Malignancies
^177^Lu-oxodotreotide/DOTATATE	PRRT	NCT04711135	Study to Evaluate Safety and Dosimetry of Lutathera in Adolescent Patients With GEP-NETs and PPGLs
^177^Lu-Octreotate	PRRT	NCT02743741	Lu-DOTATATE Treatment in Patients with ^68^Ga-DOTATATE Somatostatin Receptor Positive Neuroendocrine Tumors
^177^Lu-DOTATATE	PRRT	NCT02236910	An Open Label Registry Study of Lutetium-177 (DOTA0, TYR3) Octreotate (Lu-DOTA-TATE) Treatment in Patients with Somatostatin Receptor Positive Tumors
^177^Lu-DOTATATE	PRRT	NCT01876771	A Trial to Assess the Safety and Effectiveness of Lutetium-177 Octreotate Therapy in Neuroendocrine Tumours
^90^Y-DOTA tyr3-Octreotide	PRRT	NCT00049023	Radiolabeled Octreotide in Treating Children with Advanced or Refractory Solid Tumors
^131^I-MIBG	MIBG	NCT03015844	A Compassionate Use/Expanded Access Protocol Using ^131^I-MIBG Therapy for Patients with Refractory Neuroblastoma and Metastatic Pheochromocytoma
^131^I-MIBG	MIBG	NCT01850888	MIBG for Refractory Neuroblastoma and Pheochromocytoma
^131^I-MIBG	MIBG	NCT01590680	Expanded Access Protocol Using ^131^I-MIBG
^131^I-MIBG	MIBG	NCT01413503	A Phase II Study of ^131^I- Metaiodobenzylguanidine (MIBG) for Treatment of Metastatic or Unresectable Pheochromocytoma and Related Tumors
^131^I-MIBG	MIBG	NCT01377532	Compassionate Use of ^131^I-MIBG for Patients with Malignant Pheochromocytoma
^131^I-MIBG	MIBG	NCT00107289	Iodine I-131 Metaiodobenzylguanidine in Treating Patients with Recurrent, Progressive, or Refractory Neuroblastoma or Malignant Pheochromocytoma or Paraganglioma
Ultratrace ^131^I-MIBG	MIBG	NCT02961491	Expanded Access Program of Ultratrace Iobenguane I-131 for Malignant Relapsed/Refractory Pheochromocytoma/Paraganglioma
Tipifarnib	Farnesyltransferase inhibitor	NCT04284774	Tipifarnib for the Treatment of Advanced Solid Tumors, Lymphoma, or Histiocytic Disorders with *HRAS* Gene Alterations, a Pediatric MATCH Treatment Trial

The first medication clinicians should consider in pediatric PPGL is an α_1_-adrenergic antagonist, such as the non-competitive long-acting phenoxybenzamine or competitive short-acting drugs, typically doxazosin. Alpha-adrenergic antagonists help to reduce the symptoms associated with catecholamine excess, improve hypertension, and prepare patients for therapeutic interventions, therefore α-adrenergic antagonists should be started in all PPGL patients (1, 26). Blood pressure and heart rate should be monitored, and β-adrenergic blockade should be considered in patients who have persistent tachycardia but only after starting α-adrenergic blockade for at least 2 to 3 days to avoid the effect of unopposed α-adrenergic stimulation (16). To prevent complications related to catecholamine release during treatment, patients should start α-adrenergic blockade 7 to 14 days before surgery or before non-surgical treatments, such as chemotherapy or radiotherapy, as these may all result in catastrophic surges of stored catecholamines (26). Competitive inhibition of tyrosine hydroxylase by α-methyl-L-tyrosine (metyrosine, or Demser^®^) results in decreased synthesis of all downstream catecholamines and can be helpful for patients with highly elevated levels of catecholamines to improve hemodynamic stability before and after surgery but must be used cautiously as it is contraindicated in patients with severe depression and suicidal ideation (17, 26). Calcium channel blockers, especially amlodipine, can also be helpful in controlling hypertension in pediatric patients with PPGL (77).

Surgical interventions should be considered even for patients with metastatic disease, to reduce the tumor burden contributing to catecholamine excess and to improve radiopharmaceutical uptake in remaining metastatic lesions (26). Genetic testing preoperatively can be helpful to inform the surgical approach, as cortical-sparing adrenalectomy is preferred in younger patients to reduce the risk of adrenal insufficiency (and requirement for exogenous steroids) and does not seem to increase the risk of recurrence for patients with mutations in *VHL*, *RET*, or *NF1*, but in patients with higher risk for metastatic disease, such as those with *SDHB* mutations, more extensive resection may be needed (21).

The approach to the patient with metastatic disease depends on the extent of disease and the rate of progression. For patients with very extensive disease or rapid progression, chemotherapy should be tried first, with first-line therapy consisting of cyclophosphamide, vincristine, and dacarbazine (CVD); temozolomide as monotherapy may also be considered as an alternative or a second-line agent in these cases (78). The impact of SDH deficiency on chemoresistance, as previously mentioned, suggests that PARP inhibitors such as olaparib may be useful in combination with temozolomide, and a clinical trial is currently recruiting to evaluate this combination in adults (NCT04394858). Hypoxia-inducible factors increase expression of growth factors such as VEGF, platelet-derived growth factor (PDGF), and transforming growth factor α (TGFα), which bind to their respective receptor tyrosine kinases (53). Therefore, additional therapies to consider include tyrosine kinase inhibitors with anti-VEGF activity, such as sunitinib (53). As a last resort, additional approaches to consider include immunotherapy with checkpoint inhibitors (e.g., pembrolizumab or combination nivolumab-ipilimumab) in patients with cluster 1 mutations (consequent to impaired immune recognition related to pseudohypoxia) or mTORC1 inhibitors (e.g., everolimus) in patients with cluster 2 mutations (mTORC1 regulates cellular activity downstream of kinase signaling pathways), but additional studies are needed in children with metastatic PPGL (78).

For patients with less rapid progression or smaller tumor burden, systemic radiotherapy can be particularly useful as part of a personalized approach when lesions have avidity on imaging scans for the corresponding diagnostic radionuclides. For patients with DOTA-SSA-avid lesions, as is often seen with cluster 1 mutations, PRRT with ^177^Lu-DOTA-SSA (“hot SSA”) or SSAs that do not carry radiopharmaceuticals (referred to as “cold SSAs”) may also be helpful, especially for *SDHx* patients with DOTATATE-avid lesions, and additional clinical trials are evaluating these in children (**Table 4**). Conventional or high-specific activity (HSA) ^131^I-MIBG (referred to as Ultratrace™, or Azedra^®^) may be particularly useful for patients with cluster 2 mutations who are more likely to have adrenal disease but requires positive findings on diagnostic ^123^I-MIBG scan before use (69). When considering systemic radiotherapy, clinicians should ideally perform both ^123^I-MIBG and ^68^Ga-DOTATATE scans to determine which radiopharmaceutical best shows all the metastatic lesions in order to guide which radiotherapy should be used to treat the patient (79).

Locoregional approaches may be helpful for certain aspects of metastatic disease. External radiation therapy can be useful for head and neck primary disease due to a more favorable risk profile as compared to surgical intervention and for rapidly-growing metastatic tumors, such as in bone, to provide symptomatic relief (1, 26, 78). Treatment of a primary HNPGL by external beam radiation therapy, therefore, may be helpful for those with *SDHD* mutations. Bone metastases are also treated with bisphosphonates or denosumab (3, 26). Interventional procedures such as radiofrequency ablation, cryoablation, or ethanol injection can be used in cases for treatment of a single metastatic lesion or oligo-metastases (3, 26). Furthermore, patients with inoperable primary tumors can be considered for potential treatment with a cold-SSA if lesions are ^68^Ga-DOTATATE-avid, as reported in an adult *SDHB* patient with pterygopalatine fossa PGL, who was successfully controlled for 36 months with octreotide (80).

Belzutifan is one of the newer targeted therapies that specifically inhibits HIF-2α and has been approved to treat VHL-associated tumors (81). Given the central role that HIF-2α plays in hypoxia signaling, this therapy could be incredibly impactful for patients with cluster 1 mutations. A recent case report demonstrated the efficacy of belzutifan in treating multiple PGLs in an adolescent patient with PZS, with improvement in biochemical (normetanephrine and chromogranin A) and hematologic (erythropoietin and hemoglobin) markers and reduced tumor size on imaging. Belzutifan was well-tolerated in this patient with minimal side effects, most notably, anemia that did not require transfusion (81).

While many studies examining prognosis, disease progression, and outcomes in PPGL are focused on adults or include both adults and children, it seems that the most important prognostic factor for disease-free survival (DFS) is extent of surgical resection of the primary tumor (for pediatric PPGL: 45.6% for complete resection vs. 24.1% for incomplete resection, p < 0.001) (7, 21, 30). Redlich et al. described other significant differences in 10-year DFS in pediatric patients with PPGL, including the presence of metastatic disease (29.6% vs. 43.5% in those without metastatic disease, p = 0.014), and PGL (36.6% vs. 47.8% in PCC, p = 0.039), whereas the presence of an *SDHB* mutation was associated with lower DFS but did not reach statistical significance (45.1% in non- *SDHB* vs. 24.4% in *SDHB*, p = 0.063) (7). Outcomes in pediatric patients with *SDHB* mutations have been well-described by Jochmanova et al., and specific outcomes are also discussed above (23).

## Perspectives and Future Directions

This review highlights important features to consider in pediatric patients with PPGL, with an emphasis on metastatic disease. Pediatric PPGL is overwhelmingly due to an underlying genetic predisposition, especially from cluster 1 genes associated with pseudohypoxia. The central role of hypoxia signaling by HIF-2α in these patients results in a pro-metastatic phenotype and disruption of normal development of chromaffin cell precursors that can result in increased susceptibility to PPGL in multiple tissues. Indeed, the biochemical and secretory features could be described as more primitive or underdeveloped in pediatric patients with cluster 1 mutations predisposing them to PPGL. All pediatric PPGL patients require lifelong surveillance and monitoring, even those with cluster 2 mutations who may not present with PPGL until their teenage years. To the extent possible, these rare and challenging patients should be managed at centers of expertise with multidisciplinary teams that can address the different aspects of care.

Rather than viewing the pediatric patient with a genetic mutation as having an immutable risk factor for PPGL, the knowledge of this risk can guide clinicians to develop an appropriate screening and management plan with their patients and families, especially since the penetrance of PPGL is incomplete for all of the susceptibility genes (26). Genetic counseling can play an important supportive role in addition to contributing valuable information about risk of disease. Reducing anxiety by dispelling misconceptions about genetic risks as well as emphasizing preventive strategies and forming a screening plan to help reduce anxiety and empower patients and families is a key part of the approach to genetic counseling as shown in a study of patients with *SDHx* mutations. Out of 164 patients, only 2 (1.2%) had increased anxiety in response to genetic testing results, and only 1 (0.6%) did not proceed with any preventive measures nor any screening for her positive children (82).

Though not as prevalent a cause of metastatic disease as cluster 1 mutations, additional studies of targeted therapies for patients with cluster 2 mutations are needed in the pediatric population as our increased understanding of cellular pathways has generated specific therapeutic targets. Mweempwa et al. demonstrated efficacy of a selective RET inhibitor (selpercatinib) in metastatic PCC due to an activating gene fusion of *RET*-*SEPTIN9* in an adult with recurrent PCC with biopsy-confirmed metastatic disease to the lungs and liver with negative urinary metanephrines but highly elevated chromogranin A (33,710 µg/L). After 12 weeks of therapy with selpercatinib, chromogranin A had decreased to 598 µg/L, and pulmonary and hepatic tumors demonstrated reduction in size on CT (46% in the sum of diameters of lesions), raising the possibility that this targeted therapy may also be useful for pediatric patients with activating *RET* mutations; however additional studies are needed (83). Similarly, targeted therapy directed by the genetic background may also inform the approach to patients with NF-1. Selumetinib selectively inhibits MEK in the RAS-MAPK pathway, and in children with inoperable plexiform neurofibromas, treatment with selumetinib resulted in a reduction in tumor volume (median reduction of 27.9%) and progression-free survival of 84% (compared to 15% in natural history controls) at 3 years from treatment initiation. MEK inhibitors have also shown responses in other *NF1*-related tumors (e.g., optic pathway glioma), suggesting that PPGLs driven by *NF1* mutations might also be considered for this targeted therapy (84).

PPGLs have been described as the tumors that are the most highly genetically determined of any human tumor, and in children, around 80% have a germline predisposition. When accounting for those with additional somatic changes (e.g., *EPAS1* gain-of-function mutations), an even higher proportion is realized. Germline genetic testing to determine an underlying cause of PPGL is mandatory, especially in children with metastatic disease, but somatic tumor genetic testing has clear benefits as well and may identify new genes and targetable mechanisms that can personalize the care of these vulnerable patients.

## Author Contributions

MK drafted and revised the initial manuscript, figures, and tables. MN aided in conceptualizing and contributing to the manuscript. He also revised the manuscript. AJ aided in conceptualizing, contributing, and revising the manuscript. KP conceived, conceptualized, critically reviewed the manuscript for intellectual content, and aided in revision of the manuscript. All authors contributed to the article and approved the submitted version.

## Funding

This work was supported, by the Intramural Research Program of the National Institutes of Health, *Eunice Kennedy Shriver* National Institute of Child Health and Human Development.

## Conflict of Interest

The authors declare that the research was conducted in the absence of any commercial or financial relationships that could be construed as a potential conflict of interest.

## Publisher’s Note

All claims expressed in this article are solely those of the authors and do not necessarily represent those of their affiliated organizations, or those of the publisher, the editors and the reviewers. Any product that may be evaluated in this article, or claim that may be made by its manufacturer, is not guaranteed or endorsed by the publisher.
